# Editorial: Challenges in Posterior Circulation Ischemic Stroke

**DOI:** 10.3389/fneur.2021.789836

**Published:** 2021-11-17

**Authors:** Volker Puetz, Daniel Strbian, Thanh N. Nguyen, Simon Nagel

**Affiliations:** ^1^Department of Neurology, University Hospital Carl Gustav Carus, Technische Universität Dresden, Dresden, Germany; ^2^Dresden Neurovascular Center, University Hospital Carl Gustav Carus, Technische Universität Dresden, Dresden, Germany; ^3^Department of Neurology and Neurosciences, Helsinki University Central Hospital and University of Helsinki, Helsinki, Finland; ^4^Department of Neurology and Radiology, Boston Medical Center, Boston University School of Medicine, Boston, MA, United States; ^5^Department of Neurology, Heidelberg University Hospital, Heidelberg, Germany

**Keywords:** posterior circulation stroke, basilar artery occlusion, endovascular treatment, prognosis, anatomy

Posterior circulation ischemic stroke comprises an estimated 15–20% of all ischemic strokes and differs from anterior circulation stroke in many ways. An acute occlusion of the basilar artery can trigger one of the most devastating ischemic stroke syndromes with reduced level of consciousness, loss of brainstem reflexes and tetraplegia. However, posterior circulation stroke is frequently difficult to diagnose clinically due to varying symptoms and signs like isolated vertigo and dizziness. Knowledge about specific brainstem symptoms and syndromes can aid early clinical recognition and thus, rapid initiation of appropriate diagnostic and therapeutic approaches (1). Due to the limited diagnostic accuracy of CT-based neuroimaging in the posterior circulation, magnetic resonance imaging is the preferred method for radiological confirmation of posterior circulation ischemia, however has limited availability and can be false-negative in the hyperacute setting (2). The anatomy of the posterior circulation is highly variable and stroke syndromes within have greater etiologic variability compared to the anterior circulation. Consequently, patients with acute posterior circulation ischemia seem to respond differently to acute revascularization therapies like intravenous thrombolysis (IVT) and endovascular therapy (EVT) (3). While mechanical thrombectomy has become the new gold standard in acute recanalization therapy for anterior circulation large vessel occlusions (acLVO), its efficacy and safety in acute basilar occlusion is still under debate despite recent completion of two randomized controlled trials (4–6). In parallel to acLVO where the Alberta Stroke Program Early CT Score (ASPECTS) identifies patients with benefit from EVT, imaging may play an important role to identify patients who benefit from such therapies in posterior circulation stroke and particularly basilar artery occlusion (7–11). Moreover, while perfusion imaging can facilitate patient selection for recanalization therapies in the anterior circulation, particularly in patients with unknown or late time-window, its role and relevance in posterior circulation ischemic stroke is currently less well-described (12, 13).

In the context of this clinical and scientific background, this Research Topic covers relevant aspects of posterior circulation ischemic stroke. Three main aspects are discussed.

First, the clinical diagnosis of posterior circulation ischemic stroke is frequently difficult due to varying and non-specific symptoms. The narrative review by Hoyer and Szabo summarizes important pitfalls in its diagnosis in the prehospital and emergency department setting, and provides strategies and approaches to improve speed and accuracy of its recognition and early management. Data on anatomical variants, i.e., bilateral vertebral hypoplasia as a potential risk factor for ischemic stroke, posterior fossa venous drainage and mechanisms of posterior circulation blood flow regulation and its implications for posterior circulation stroke are detailed in the articles by Hsu et al., De Miquel, and Tamayo and Siepmann, respectively.

Second, several articles of the collection cover novel aspects of acute treatment in patients with posterior circulation ischemic stroke and particularly basilar artery occlusion. Known data for IVT in late or unknown time window mostly focus on patients with anterior circulation ischemic stroke. It is therefore reassuring that the safety and efficacy of IVT in this scenario seem similar in patients with posterior circulation ischemic stroke, as addressed in the article by Macha et al. Their findings may inform clinicians in the usage of alteplase beyond 4.5 h from symptom onset in selected patients with posterior circulation ischemic stroke. Scientifically, however, more data on this topic is needed until a general recommendation can be made.

Given the unproven benefit of EVT on improved functional outcome in patients with basilar artery occlusion, the meta-analysis by Mbroh et al. suggests that EVT in posterior circulation large vessel occlusion (pcLVO) may be comparably sufficient in obtaining favorable functional outcome compared with acLVO. These findings parallel results of the recently published BASILAR registry, where patients who received EVT within 24 h after the estimated time of basilar artery occlusion had an improved chance to achieve a favorable functional outcome compared to patients who received best medical management only (14). In contrast, in the analysis of State-wide stroke registry data by Gruber et al., additional EVT was not superior compared to best medical management alone in patients with acute basilar artery occlusion. One must keep in mind that the results of the above mentioned analyses originate from non-randomized studies and are therefore limited in their informative value.

Patients with acute basilar artery occlusion who present with coma are unlikely to have a good clinical outcome. However, Ritvonen et al. demonstrate in their study that one fifth (21/103, 20.4%) of these patients can still achieve a favorable functional outcome. Moreover, recanalization and a lesser extent of early ischemic changes on neuroimaging [i.e., posterior circulation Acute Stroke Prognosis Early CT Score (pc-ASPECTS) > 8] were associated with favorable outcome in this study. These results confirm findings from a previous analysis of the BASICS registry data and underline the prognostic importance of imaging in patients with basilar artery occlusion (9, 15, 16).

As EVT is available in specialized stroke centers, patients with basilar artery occlusion are frequently transferred from remote hospitals in a drip-and-ship approach. The article by Alemseged and Campbell summarizes current data on tenecteplase as an alternative thrombolytic agent with greater fibrin specificity and longer half-life compared with alteplase in this scenario. As they recently demonstrated, tenecteplase achieved higher reperfusion rates in patients with large vessel occlusion including basilar artery occlusion (17, 18).

The effectiveness of EVT in acLVO has primarily been shown for patients with ICA or M1-segment occlusions, but is now also frequently performed in patients with more distal (e.g., M2-segment) occlusions. Whether EVT is safe and effective in patients with posterior cerebral artery (PCA) occlusion has been analyzed in the article by Herweh et al. as a collaboration of four major stroke centers. Their main conclusion was that EVT in isolated posterior cerebral artery occlusion appears safe and at least immediately effective, however further data from prospective or randomized studies are needed, especially on the longterm outcome activities of daily living. EVT for fetal PCA thrombectomy is another posterior circulation frontier that is also being explored in patients presenting with disabling stroke (19). Lastly, Kaiser et al. show that a regular thrombus surface (defined as smoothly straight, convex, or concave) is associated with a higher chance for successful first pass reperfusion in patients with acute basilar artery occlusion which confirms previous findings in patients with acLVO (20).

Third, as outlined above, the stroke etiology in patients with basilar artery occlusion seems more heterogenous compared to acLVO. Artery-to-artery embolism and intracranial atherosclerotic disease play an important role. Characterization of such stenoses is important for acute treatment decision making and to tailor therapy in secondary stroke prevention. By using 3T high-resolution magnetic resonance imaging in patients with recent posterior circulation stroke due to intracranial vertebrobasilar atherosclerotic disease with 70–99% stenosis, Hou et al. demonstrated that intraplaque enhancement and vertebral artery involvement were associated with artery-to-artery embolism. Questions arise whether these patients may benefit from more aggressive antiplatelet regimens.

In conclusion, this issue of *Frontiers in Neurology* provides an integrated overview of the hot topics in the field of posterior circulation ischemic stroke. Emerging from the article collection is a complex picture with focus on the anatomical, clinical and pathophysiological correlates of posterior circulation stroke, acute treatment including intravenous and endovascular therapies. The studies published in this issue emphasize the need for further research to better delineate pathophysiological aspects, clinical recognition and treatment decision making in these patients, in the wake of the BASICS and BEST basilar artery occlusion trials (4, 5, 21). Stratification of patient selection by severity of disease, imaging including multimodal CT or MRI, introduction of novel thrombolytics (i.e., tenecteplase) may play an important role in modifying the natural history of posterior circulation stroke. We look forward to learning the results of this research in the future.

## Author Contributions

VP drafted the manuscript. DS, TN, and SN revised the manuscript for intellectual content. All authors contributed to the article and approved the submitted version.

## Conflict of Interest

The authors declare that the research was conducted in the absence of any commercial or financial relationships that could be construed as a potential conflict of interest.

## Publisher's Note

All claims expressed in this article are solely those of the authors and do not necessarily represent those of their affiliated organizations, or those of the publisher, the editors and the reviewers. Any product that may be evaluated in this article, or claim that may be made by its manufacturer, is not guaranteed or endorsed by the publisher.
